# News and Coming Events

**Published:** 1907-04-06

**Authors:** 


### NEWS AND COMING EVENTS.

Dr. F. M. Sandwith has been appointed to the Gresham Lectureship, in place of the late Dr. Symes Thompson.

The Congress of Russian Surgeons will be held in Moscow on December 28-30 next, under the presidency of Professor Esapjesko, of Odessa.

The annual dinner in aid of the National Sanatorium for Workers suffering from Consumption will be held at the Hotel Cecil on May 14. H.R.H. the Prince of Wales will preside.

Thirty-seven female candidates presented themselves for examination for admission to the M.D. degree recently at the University of Moscow, and all succeeded in satisfying the examiners.

The sixth International Congress of Dermatology will be held in New York in September next, Dr. James White, of Boston, presiding. The general secretary of the Congress is Dr. Fordyce.

The University of Glasgow has instituted a Lectureship in Applied Anatomy, and has appointed Dr. Robert Kennedy, M.A., D.Sc., to be the lecturer. Dr. Kennedy was at one time George A. Clarke Scholar in the University, and is at present an assistant-surgeon to the Western Infirmary.

At the annual meeting of the Royal Eye Hospital, Southwark, on the motion of Sir William Collins, M.P., the governors resolved to refer to the architect the question of the practicability of enlarging the out-patient and in-patient departments on the present hospital site, regard being had to the requirements of the nursing staff. There would seem to be little, if any, limit to the possibilities of indefinitely increasing the accommodation provided by ophthalmic hospitals in the Metropolis for the free relief of patients who seek such aid at these institutions.

The new Somerset Hospital at Cape Town is in the throes of a crisis. Recently the Board decided to have three medical residents instead of four. The resident surgeon, Dr. Moffat, has resigned, and the matron has followed his example. After a somewhat stormy discussion, the Board decided to accept the resignation of the resident surgeon, and to refer that of the matron to the House Committee to deal with. The appointment of resident surgeon is to be advertised at a salary of £450, with house, but some of the Board appear to be in favour of the appointment of an administrative medical superintendent.

The yearly report of the After-care Association for poor persons discharged recovered from asylums for the insane shows that this excellent organisation is continuing its good work with very encouraging results. During the year 308 cases were dealt with, and in the majority the committee was able to find suitable employment for applicants, though a few cases had to be declined as the applicants were quite unable to bear the strain of even ordinary occupation. The Association is in need of funds, its income, derived entirely from voluntary contributions, being only £972. It investigates all cases brought to its notice very thoroughly, and the expense of such systematic investigation is considerable. The report shows that it has a large number of old and tried friends, and the account of the good work it has done during the past year ought to bring it many new friends.

Sir R. Douglas Powell has been re-elected President of the Royal College of Physicians.

A Congress of practitioners will be held in Paris this month, with a view of considering certain necessary reforms in the French medical curriculum.

The German Anatomical Society will hold its twenty-first annual meeting at Wurzburg on April 24-27, under the presidency of Professor Romiti, of Pisa.

The annual meeting of the Humanitarian League will be held in the Essex Hall, Essex Street, Strand, on Friday evening, April 19, Mr. G. Greenwood, M.P., in the chair.

Dr. Weber, assistant-physician in an asylum at Hofheim, in Germany, was shot at by one of the inmates while going his rounds. The patient, a paranoic, who had obtained a revolver from somewhere, fired at the doctor and inflicted three wounds, which proved fatal.

Mr. H. C. Staniland Smith, the London representative of the Seamen's Hospital Society, Greenwich, has been appointed Secretary of the Evelina Hospital for Sick Children. Mr. Smith joined the staff of the Seamen's Hospital Society in the year 1902, where he is credited with good work, and we hope he may do much for the Evelina Hospital.

"If my son is suffering from such a valuable disease, he had better keep it." This was stated at a recent meeting of the Lancashire County Council to be the reply of the mother of a miner with ankylostomiasis, who was offered £1 a week to remain in Wigan Hospital in order that the doctors might watch his case. The disease, it is worthy to note, is one which is included in the Schedule of the Workmen's Compensation Act.

On Saturday, April 6, the doors of the Agricultural Hall, London, will open on Cordingley's Motor-car Show and the Aero Club Display, which promises to be one of the most instructive exhibitions yet held in connection with modern developments of locomotion on land and in air. In the aero section, which is under the auspices of the Aero Club, models for the large prizes offered to inventors will be on view. The exhibition will remain open till the 13th, and on Wednesday, the 10th, the Ladies' Automobile Club will hold a fashionable reunion.

Cramer, working at Munich, has published an account of two cases in which he attempted to transplant the ovaries. Experimental transplantation of these organs in lower animals has been attended with encouraging success, and some time ago Professor Knauer published a case in which it had been tried on a patient suffering from uterine atrophy. Cramer's patient was a woman with advanced atrophy of both ovaries and with a uterus that could scarcely be felt bimanually. One ovary was split, and a healthy ovary, obtained from an osteomalacic patient in the same ward and on the same day, was placed in the section. The graft was fixed in position with thin catgut sutures, strict aseptic precautions being observed. The wound healed by primary intention, and the patient made a good recovery. The uterus increased in size, and the woman menstruated twice. Then she had a short period of amenorrhœa, followed by abortion. In a second case the attempt at transplantation failed, the engrafted ovary undergoing complete atrophy.

